# A case report of Ggeneralized uterine arteriovenous malformation after molar pregnancy in an infertile woman

**Published:** 2018-02

**Authors:** Firoozeh Ahmadi, Somayeh Moukhah

**Affiliations:** *Department of Reproductive Imaging, Reproductive Biomedicine Research Center, Royan Institute for Reproductive Biomedicine, ACECR, Tehran, Iran. *

**Keywords:** Color doppler ultrasonography, Embolization, Molar pregnancy, Arteriovenous malformation

## Abstract

**Background:**

Uterine arteriovenous malformation (UAVM) is a rare vascular condition in reproductive age presented mostly with bleeding. Although this malformation is infrequent, it is potentially life-threatening. Transvaginal Doppler ultrasonography is a widely available, noninvasive and excellent diagnostic method.

**Case:**

The case is a 30-yr-old woman with a history of eight-yr infertility.following intrauterine insemination treatment, she had a molar pregnancy. Despite methotrexate treatment, there was persistent vaginal bleeding. Assessment of this patient was done with transvaginal sonography and color Doppler. According to suspicious appearances, angiography was planned for confirmation of UAVM.

**Conclusion:**

UAVM is one of the molar pregnancy complications. The first step for diagnosis of UAVM is transvaginal ultrasonography and color Doppler assessment. Embolization is the best treatment for women who intend to preserve fertility.

## Introduction

Uterine arteriovenous malformation (UAVM) is known as a direct connection of the arterial system to the venous system, without contribution of capillary vessels (1). This vascular abnormality is rare as the review of literature shows the incidence UAVM is 0.10%. The first case was presented in 1926 (2). This malformation is considered as local or generalized lesions. UAVM classifies to congenital and acquired. Occurrence of acquired UAVM is more common than congenital type that resulted from some situations such as “gestational trophoblastic disease, pelvic trauma, surgical procedures (cesarean section, curettage), cervical or endometrial carcinoma, infection and exposure to diethylstilbestrol” (3).

Nowadays, diagnosis of UAVM is possible with noninvasive methods including transvaginal ultrasound and color Doppler. Although transvaginal ultrasound makes with better quality and be considered as the first step for assessment of women with vaginal bleeding, these ultrasound images are nonspecific and assist commonly to the differential diagnosis of UAVM from the other conditions such as gestational trophoblastic disease and retained product of conception (4, 5). it was shown that color Doppler is a preferable and confident method for initial diagnosis of UAVM (6-8). Therefore, it is suggested to confirmation of transvaginal ultrasound finding with color Doppler for the increased accuracy (4, 5). However, angiography an invasive method for diagnosis of UAVM. It is a gold standard for definitive detection of UAVM (5, 9). Embolization is the best treatment for women who intend to preserve fertility.

In the current case report, the pregnant woman with a history of infertility who developed UAVM following a molar pregnancy is presented. Oral consent had been obtained from the patient before the interview.

## Case report

**Figure 1:**
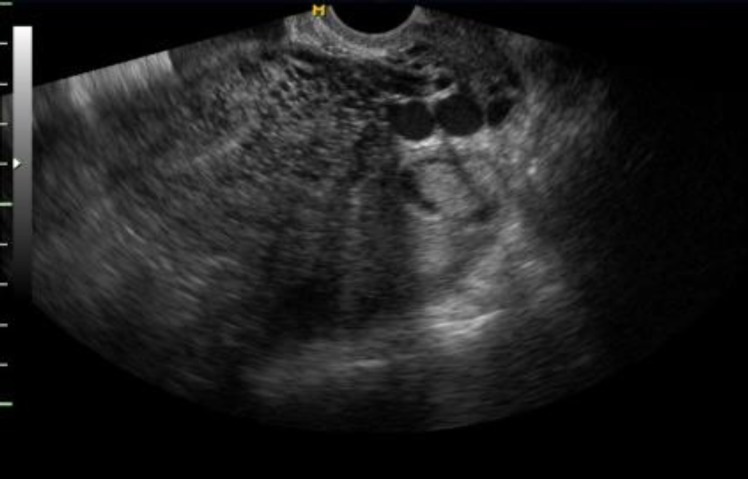
Transverse transvaginal ultrasound (TVUS) shows multiple small anechoic spaces in the anterior myometrium producing a "spongy" myometrial echotexture

In February 2008, a 30-yr-old woman, presented at the infertility clinic of Reproductive Biomedicine Research Center of Royan Institute (Tehran, Iran). Her chief complaint was 8-yr of old infertility with tubal factor and also vaginal bleeding. In obstetrics and gynecologic history, she had done laparoscopic operation 3 yr ago for endometriosis treatment. Moreover, intrauterine insemination was done that resulted in a GTD and methotrexate was prescribed for her but due to severe bleeding the patient underwent three curettages. She had uterine bleeding with negative beta- Human chorionic gonadotropin levels, therefore transvaginal sonography was done and we detected numerous echo-free tubular spaces in anterior myometrium("spongy" myometrium echo texture)with normal endometrium (Figure 1). 

**Figure 2:**
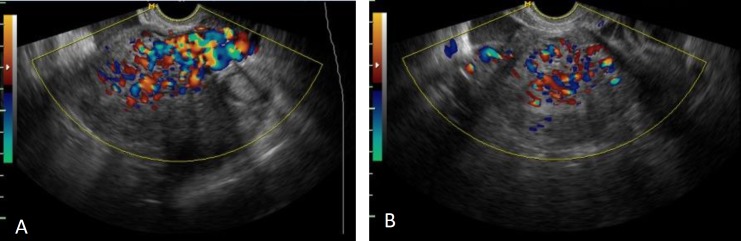
A (Sagittal view) and 2 B (Transverse view). Color Doppler ultrasound in this case shows a mosaic flow within the tubular structure with color aliasing

**Figure 3:**
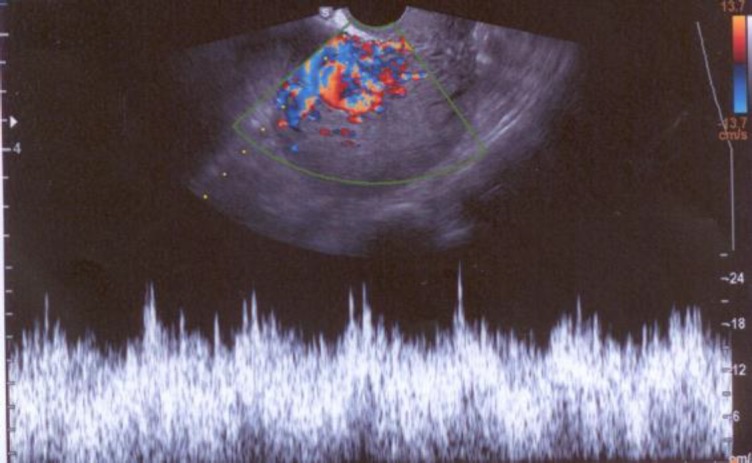
Spectral Doppler ultrasound shows high velocity and low-resistance flow with little variation between systolic and diastolic velocities, compatible with a uterine AVM

**Figure 4:**
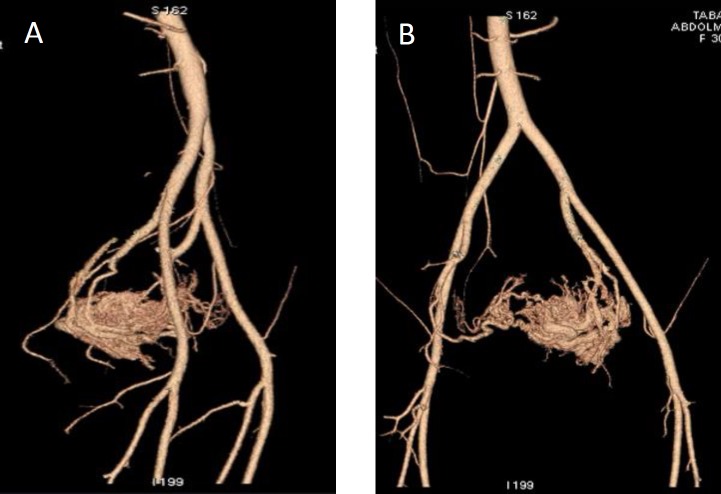
A, B. Enlargement of right uterine arteries has been showed and all typical angiographic findings of uterine confirmed AVMs.

According to these suspicious appearances, color Doppler was done and mosaic patternsof color signals whit in myometrium cystic spaces with high-velocity flow was seen and all cystic spaces fill with flow (Figure 2A, 2B, 3). These features were suspected us to UAVM and angiography approved it (Figure 4A, 4B). Since the patient desired to preserve her fertility, embolization was done and following in vitro fertilization (IVF) procedure she got pregnant and terminated successfully pregnancy that resulted to birth a girl baby in 5 yr ago. 

Albeit embolization, vaginal bleeding continued. After transvaginal ultrasound and also color Doppler assessments the UAVM was persistent. The remarkable note is that although embolization was done for fifth times during 7 yr ago and also the UAVM was durable, the patient has got pregnant afterward the second time IVF and this pregnancy resulted to birth a boy baby with weight 2,650 gr.

## Discussion

UAVM has various clinical presentations from an asymptomatic patient to life-threatening condition, but the most prevalent symptom is periodical and severe vaginal bleeding that often occurs in the reproductive age of a woman (10, 11). We reported an acquired UAVM that was presented with genital bleeding following the gestational trophoblastic disease. Farias *et al* presented a similar case with abnormal genital bleeding (12). Considering that ultrasonography is the first-step for imaging assessment in most studies, we planned a transvaginal sonography (12-14). Commonly UAVM appearance in grayscale sonography contains an ill-defined and homogeneous mass with multiple myometrial and endometrial hypoechoic cystic or tubular-like structures of varying sizes (15). 

In our ultrasonography assessment, we found the mix echo pattern with several echo-free cystic lesions in the anterior myometrium. These sonographic features are seen in the other diseases such as gestational trophoblastic disease, retained conception products. This finding is supported in other studies (4, 12, 15, 16). In color Doppler assessment of UAVM, a cystic or tubular space has vascular nature with the low-resistance and high-velocity stream (15). The accurate diagnosis of these lesions is angiography of pelvic (2). Although the role of MRI was emphasized in Farias *et al* study, MRI is an expensive and time-consuming method. Despite MRI finding, confirmation of angiography is necessary for treatment of UAVM.

There are different kinds of methods for management of UAVM based on the age of women, desire for future fertility and severity of bleeding such as hysterectomy, surgical removal of AVM, laparoscopic bipolar coagulation, medical therapy with combined oral contraceptives and embolization (17). To aggravate the bleeding occurrence, curettage is not a suitable management for UAVM therapy. For the woman in the reproductive age and intended to preserve fertility, the best option for treatment is embolization.

## Conclusion

Nowadays, the first step for diagnosis of UAVM is noninvasive methods such as transvaginal ultrasound and color Doppler, but angiography is an invasive method for diagnosis of UAVM. In addition, it is a gold standard for definitive detection of UAVM. Embolization is the best treatment for women who intend to preserve fertility. The remarkable note is that although embolization was done for several times, the UAVM was durable and this woman is pregnant currently afterward the second-time IVF was done. 
